# Application of SrFeO_3_ perovskite as electrode material for supercapacitor and investigation of Co-doping effect on the B-site

**DOI:** 10.55730/1300-0527.3475

**Published:** 2022-09-01

**Authors:** Mohammad AHANGARI, Elham MAHMOODI, Nagihan DELİBAŞ, Jafar MOSTAFAEI, Elnaz ASGHARI, Aligholi NIAEI

**Affiliations:** 1 Department of Chemical and Petroleum Engineering, University of Tabriz, Tabriz, Iran; 2 Department of Physics, Faculty of Art and Science, of Sakarya University, Sakarya, Turkey; 3 Department of Physical Chemistry, Faculty of Chemistry, University of Tabriz, Tabriz, Iran

**Keywords:** Supercapacitor, perovskite, oxygen vacancy, partial substitution, electrochemical performance, electrode materials

## Abstract

Energy storage by supercapacitors with short charging time and high power density is one of the types of electrochemical storage systems. Perovskite oxides have been significantly investigated as promising materials for energy storage in electrochemical systems. In this study, three perovskites, SrFeO_3_, SrCoO_3_, and SrCo_0.5_Fe_0.5_O_3_, were prepared using the sol-gel method and used as supercapacitor electrode materials. In fact, in this research, two consecutive elements (Fe, Co) from the periodic table that differ by one unit in atomic number are placed in the perovskite structure to study their electrochemical properties for use in supercapacitors. From the obtained results, it was found that Co doping with a ratio of 1/1 (Co/Fe) at B site of SrFeO_3_ reduced the specific capacitance from 101.687 F g^−1^ to 60.912 F g^−1^ at a scan rate of 10 mV s^−1^. Also, the specific capacitance of SrCoO_3_ decreased from 68.639 F g^−1^ to 60.912 F g^−1^ at the same substitution rate at B site.

## 1. Introduction

With the development of industrialization, followed by an increase in environmental pollution, the production and storage of energy from renewable sources have become necessary [1–4]. In recent years, supercapacitors have been studied by many researchers due to their high energy storage performance [5–9]. Compared to batteries, these devices have high power density and good cyclic stability. They also have a higher energy density than ordinary dielectric capacitors to fill the gap between the batteries and capacitors [10–12]. Supercapacitors fall into two categories: two-layer electrical capacitors (EDLCs) and pseudocapacitors. Energy storage in EDLCs does not involve any Faraday reactions, rather, this is done through ion exchange at the electrode/electrolyte interface. An example of an electrode material used in EDLCs could be high specific surface carbon materials. Instead, pseudocapacitors store energy on the surface of conductive polymeric materials or metal oxides through Faraday reversible reactions [13–16]. Transition metal oxides are usually more stable than carbon-based materials, and have a higher energy density than conductive polymeric materials, so they are good candidates for supercapacitor electrodes [17].

Nowadays, inorganic nanoparticles have found a special place in electrochemistry applications and are widely considered by researchers. For example, the composites of Fe@Fe_2_O_3_ core-shell nanowires [18], iron minerals (magnetite, siderite, hematite, limonite and pyrite) [19] loaded on graphene respectively as a cathode in the electrofenton process to remove the drug sulfasalazine from aqueous solution. And elimination of paclitaxel as an antineoplastic drug were studied. Also, carbon nanotubes (CNTs) and double-layer CuFe nanolayer hydroxide (NLDH) [20] have been investigated for the decomposition and mineralization of the antibiotic cefazolin during the electrofenton process. Recently, these materials have been studied by researchers as electrode materials for supercapacitors.

Perovskite materials with ABO_3_ structure have also been considered due to their stable structure, high concentration of oxygen vacancy and excellent capacitance [21]. Perovskite oxides ideally have the closed formula ABO_3_ which have a cubic or almost cubic structure. It is possible to presence of ions in different sizes and valances in the A and B site of perovskite structure which shows the high flexibility of these compounds. In the cubic structure, each B site cation bonds with 6 oxygen atoms to form an octagonal (BO_6_) and each A site cation bonds with 12 oxygen atoms to obtain a stabilized perovskite structure [22,23]. In fact, the main controller of electrochemical properties of perovskite can be considered related to BO_6_ in the structure of perovskite. In addition, the physicochemical properties of perovskites can be improved by substitution at the A and B sites with another cation with different radii and valence [24–28]. One of the properties of perovskites that lead us to study them as supercapacitor electrode is their ionic and electronic conductivity [29–35]. In 2014, Mefford et al. [36] investigated the storage of anionic charge in the LaMnO3 perovskite as a supercapacitor electrode. For the first time, they used oxygen intercalation for rapid energy storage. Zhu et al. [37] synthesized SrCo_0.9_Nb_0.1_O_3-δ_ for use in supercapacitor as electrode material. Their results showed that in this type of perovskite oxide, when the discharge rate increases from 0.1 to 10 A g^−1^, the storage capacitance will be more than 92%.

In this study, the electrochemical properties of SrCoO_3_, SrFeO_3_, SrFe_0.5_Co_0.5_O_3_ perovskites as supercapacitor electrodes were investigated to evaluate the effect of partial substitution at the B-site of SrFeO_3_ and SrCoO_3_ on the performance and specific capacitance of the supercapacitor electrode. As far as we know, it is may be the first time that Co and Fe elements, which differ by one unit in atomic number, are placed in the B site of the perovskite structure, and their electrochemical properties are studied as supercapacitor electrodes. The electrochemical activity of SrFeO_3_, SrCoO_3_ and SrFe_0.5_Co_0.5_O_3_ was determined by cyclic voltametric (CV), galvanostatic charge-discharge (GCD), and electrochemical impedance spectroscopy (EIS) analyses.

## 2. Materials and methods

### 2.1. Synthesis and characterization

Raw materials include Strontium nitrate (Sr (NO_3_)_2_, assay 98.0%, Samchun, South Korea), cobalt (II) nitrate hexahydrate (Co (NO_3_)_2_.6H_2_O, assay 97%, Merck), iron (III) nitrate nonahydrate (Fe (NO_3_)_3_.9H_2_O, assay 99%, Merck, Germany), glycine (H_2_NCH_2_COOH, assay 99.0%, Samchun, South Korea). The SrFeO_3_, SrCoO_3_, and SrFe_0.5_Co_0.5_O_3_ were synthesized by the combustion sol-gel method. The preliminary materials Sr(NO_3_)_3_, Co(NO_3_)_3_.4H_2_O, and Fe(NO_3_)_3_.9H_2_O were mixed in stoichiometric ratio (to produce 1 g of SrFeO_3_, each raw material was used: 1.105, 0, and 2.11 g, respectively. Also for SrCoO_3_: 1.11, 1.32, and 0 g and for SrFe_0.5_Co_0.5_O_3_: 1.108, 0.657, and 1.057 g of the mentioned raw materials were used, respectively.) at 60 °C in 50 mL deionized water, then glycine with a 5:1 molar ratio (glycine/perovskite; about 1.96 g of glycine is needed to produce 1 g of each of the three mentioned perovskite samples) added to the solution at 80 °C. By continued heating and stirring, the gel was formed, and by auto-combustion, a black-brown perovskite powder was obtained.

Crystalline phases were identified by X-ray diffraction (Tongda TD-3700, China) and Cu Kα radiation (λ = 1.5406 Å). Diffractograms were recorded with a step of 0.02 degree per 0.5 s for 2θ between 10 and 80. The synthesized materials were morphologically investigated by scanning electron microscopy (FE-SEM, model MIRA3-TESCAN, Czech) analysis. Further analysis of the structure was carried out by energy dispersive X-ray spectroscopy (EDX, MIRA3 FEG-SEM, Tescan, Czech Republic) analyzes and elemental mapping. The pore size distribution of samples was determined by Brunauer-Emmett-Teller (BET) through nitrogen adsorption-desorption analyzer (Micromeritics, TriStar II 3020).

### 2.2. Electrochemical measurement

The electrochemical evaluations were conducted using an Autolab, PGSTAT30 Potentiostat-Galvanostat. All electrochemical analysis and measurements were done in a symmetric two-electrode system. In which copper plates were used as current collectors and paper impregnated with 1 mol/L KOH (as electrolyte material) was used as separator. Cyclic voltammetry (CV) curves were obtained at various potential scan rates between −0.8 and (+1.0) V. Electrochemical impedance spectrometry (EIS) measurements were carried out at open circuit potential (OCP) in a frequency range of 100 kHz – 10 mHz. Galvanostatic charge-discharge (GCD) tests were performed in a voltage range of −0.8 to 1.0 V with different current densities. Using CV curves and GCD curves, the specific capacitance (Cs, F g^−1^) of perovskite compounds can be obtained from Equations (1) and (2), respectively [4]:


(1) $$C_{s}=\frac{1}{2\times m\times v\times \Delta V}\int_{V_{a}}^{V_{c}}i_{\left(V\right)}dV$$

(2) $$C_{s}=\frac{i\times \Delta t}{m\times \Delta V},$$

where (A) is the current, (g) is the mass of electrode material, (s) is the discharge time, (mV s^−1^) is the scan rate, and (V) is the potential sweep window.

The energy density (E, Wh kg^−1^) and power density (P, W kg^−1^) of the supercapacitor were also obtained from the GCD curves according to Equations (3) and (4) [4, 38]:


(3) $$E=\frac{1}{2}C_{s}V^{2}$$

(4) $$P=3600\times \frac{E}{\Delta t},\text{w}$$

where (F g^−1^) is the specific capacitance, (V) is the potential drop.

## 3. Results and discussion

### 3.1. Crystal structure and morphology

**Figure 1:**
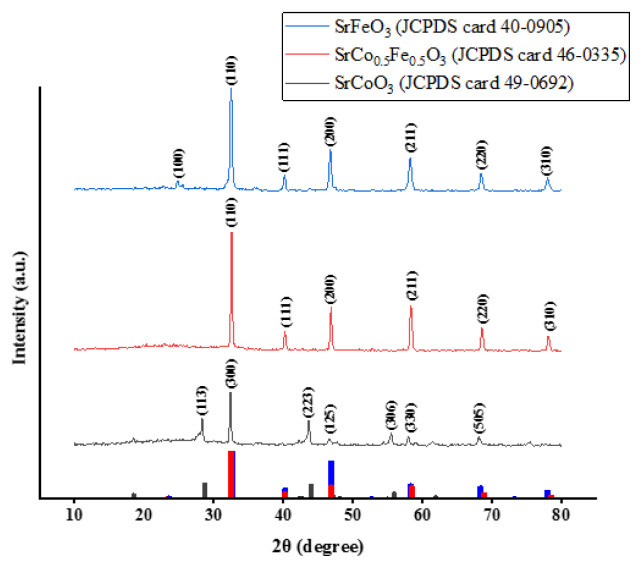
XRD pattern of SrFeo_3_, SrCo_0.5_Fe_0.5_O_3_, SrCoO_3_.

Figure 1 shows the XRD pattern of SrCoO_3_, SrFeO_3_, and SrFe_0.5_Co_0.5_O_3_ powders. From the obtained diffraction patterns, the peak sharpness of SrCoO_3_, SrFeO_3_, and SrFe_0.5_Co_0.5_O_3_ occurred at angles (2θ) of 32.655°, 32.816°, and 32.803°, respectively. This showed the sharp peak of the SF shifted slightly to the left relative to the sharp peak of the SrFe_0.5_Co_0.5_O_3_ while the sharp peak of the SrCoO_3_ shifted to the right, indicating that the SrCoO_3_ is distorted in structure of perovskite [18]. The reason for the observed shift in the XRD patterns of the synthesized samples is the difference in the elements in B site of the perovskite structure; because the oxidation states of each of the elements are different and these oxidation states directly affect the chemical bonds in the perovskite structure and cause structural differences among them. The oxygen vacancy of the perovskite structure, which is related to the oxidation states of the B site element, can be determined to some extent by the same structural differences observed from the XRD analysis of the samples. According to the obtained XRD results and comparing with the related standard XRD cards, the presence of oxygen vacancy in the structure of synthesized perovskites was confirmed. According to the obtained XRD diffraction for SrCoO_3_ structure of this perovskite has a rhombohedral structure and its diffraction pattern according to Figure 1 is very close to SrCoO_x_ (JCPDS card 49-0692), and after doping Fe along with Co (SrFe_0.5_Co_0.5_O_3_), the structure has changed to cubic (JCPDS card 46-0335). While the structure of SrFeO is cubic, its diffraction pattern is close to SrFeO_2.97_ (JCPDS card 40-0905).

**Figure 2:**
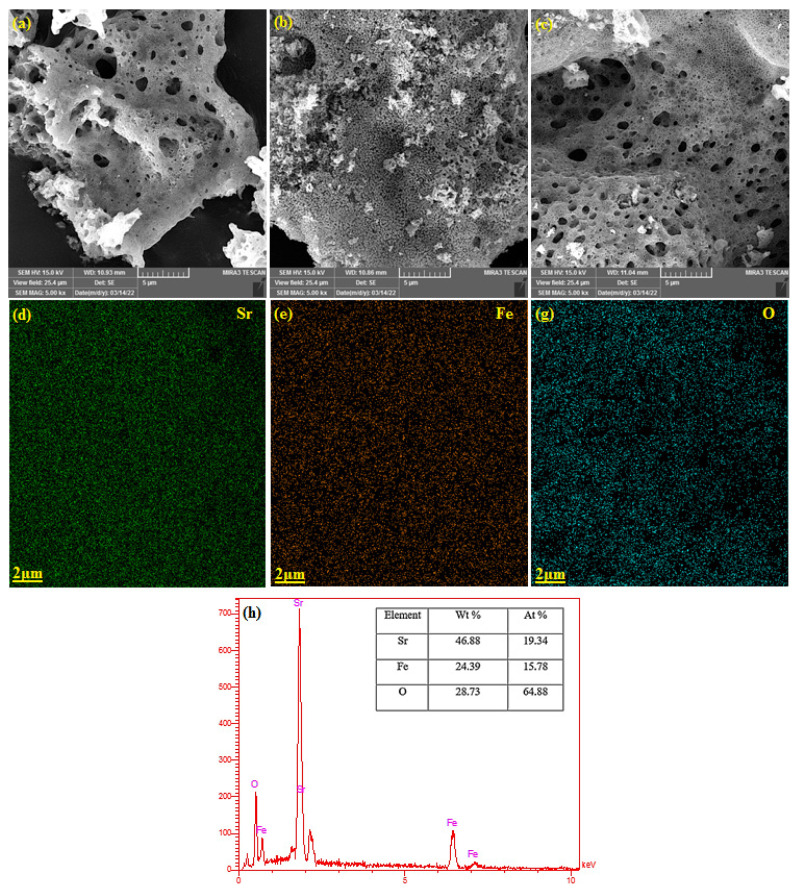
SEM image of (a) SrFeO_3_, (b) SrCoO_3_, (c) SrCo_0.5_Fe_0.5_O_3_ perovskite oxide. ((d)–(g)) The elemental mapping of SrFeO_3_. (h) EDS spectrum of sample SrFeO_3_.

**Figure 3:**
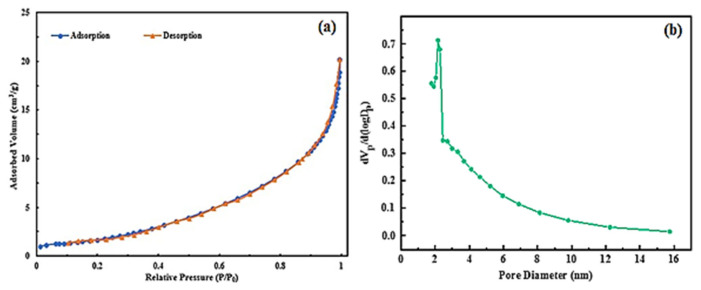
(a) N_2_ adsorption-desorption isotherm of SrCoO_3_, (b) Pore size distribution of SrCoO_3_.

The morphological structure of the synthesized perovskite compounds was obtained by SEM analysis, as shown in Figures 2a–2c. All of the synthesized perovskites have shown rough and highly porous structures that may result in an increased active surface area for the electrodes. More homogenous distribution of the pores is obvious for SrFe_0.5_Co_0.5_O_3_ compared to SrFeO_3_. Both SrCoO_3_ and SrFe_0.5_Co_0.5_O_3_ samples have also indicated small average pore sizes. These characteristics are expected to be critical factors for their supercapacitive activities. Also, analysis of nitrogen adsorption and desorption measurements was performed to obtain detailed information about the porosity nature of the synthesized samples. As shown in Figure 3 most of the pores were in the size range of 5 to 17 nm. In addition, the elemental mapping of the SrFeO_3_ composition in Figures 2d–2g shows that the distribution of Sr, Fe and O elements on the SrFeO_3_ surface is uniform, which confirms the success of SF synthesis. SrFeO_3_ was also analyzed by energy dispersive X-ray (EDX). According to the EDX results as shown in Figure 2h, the molar ratios of Sr, Fe, O are 19.34, 15.78, 64.88, which is in good agreement with the theoretical values.

### 3.2. Electrochemical performance

The oxygen vacancy sites in the structure of perovskites can provide diffusion pathways for electrolyte ions. Because the compounds are synthesized in porous form, electrolyte transport is more efficient for redox reactions during the Faraday charge storage process. What is important in charge storage in this way is the mobility of oxygen vacancy space as a charge carrier. The concentration of oxygen vacancy also depends on the structure of the perovskite. In addition, the oxidation states of the B site and the ease of access to these oxidation states have a great effect on the specific capacitance (and oxygen vacancy) [39,40].

**Figure 4:**
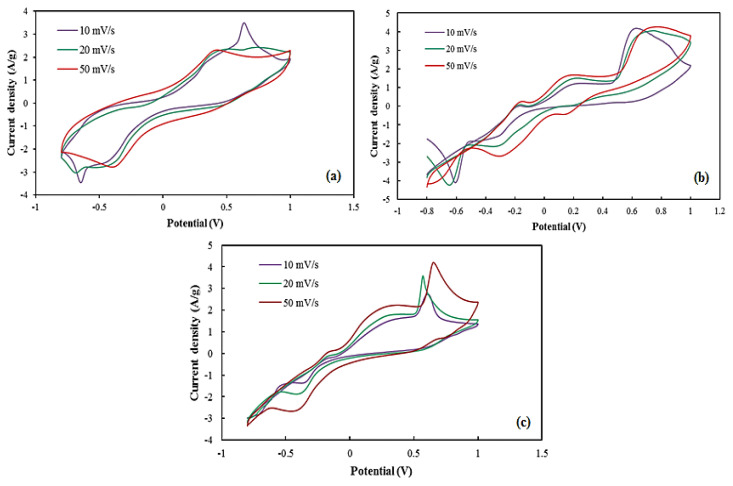
CV curves of (a) SrFeO_3_, (b) SrCoO_3_, and (c) SrCo_0.5_Fe_0.5_O_3_ electrodes at different scan rates.

CV and GCD analysis were performed to clarify perovskite oxides’ charge storage capacitance and stability, and to evaluate their specific capacitance. Figure 4 shows the CV curves of SrCoO_3_, SrFe_0.5_Co_0.5_O_3_, SrFeO_3_ with distinct redox peaks showing typical pseudo-capacitive properties. SrCoO_3_ and SrFeO_3_ capacitance can be attributed to Co^3+^/Co^2+^ and Fe^3+^/Fe^2+^ redox reactions, respectively [41,22]. As the scan rate increases, it is observed that the area related to the CV curves increases and as a result, the specific capacitance decreases.

The decrease in specific capacitance is visible by increasing the scan rate. At lower scan rates, more electrolyte ions can react electrochemically with the active material on the electrode surface, as a result, the specific capacity increases. The specific capacitance of the synthesized perovskite compounds was calculated using the CV curve by Equation (1), the results of which are given in Table 1.

**Figure 5:**
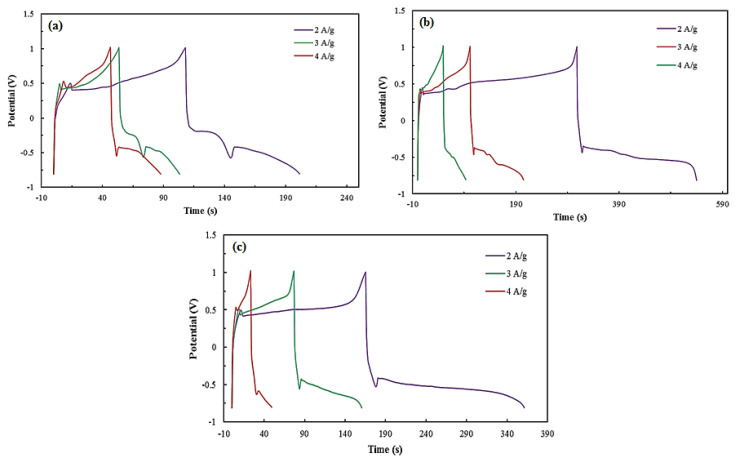
GCD curves of (a) SrFeO_3_, (b) SrCoO_3_, and (c) SrCo_0.5_Fe_0.5_O_3_ electrodes at different current densities.

**Figure 6:**
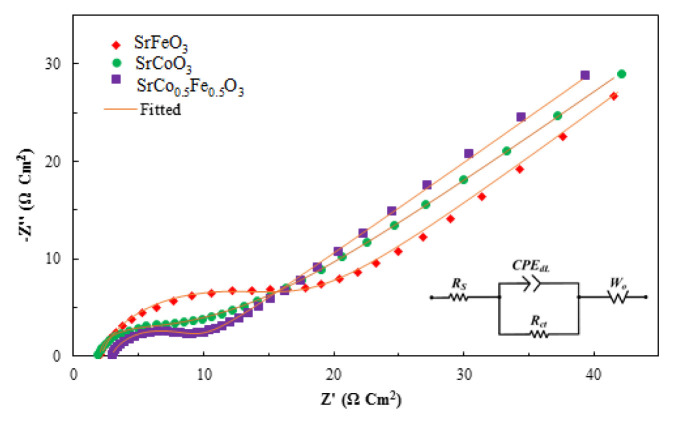
Electrochemical impedance spectra of SrCoO_3_, SrCo_0.5_Fe_0.5_O_3_, and SrFeO_3_.

The GCD curves of the SrCoO_3_, SrFeO_3_ and SrFe_0.5_Co_0.5_O_3_ electrodes at different current densities are shown in Figure 5. Table 2 contains information on the specific capacitance of perovskite compounds calculated from Equation 2. In addition, EIS analysis was performed in the frequency range from 100 kHz to 10 mHz. The results are shown in Figure 6. R_ct_ shows the resistance of the transfer of ions at the interface of the electrode/electrolyte materials. The straight line also shows the diffusion resistance of electrolyte ions in the pores of the electrode material [42,43]. At high frequencies, the presence of a small semicircle indicates the low charge transfer resistance (R_ct_) at the electrode/electrolyte interface and the rapid charge propagation between the electrolyte and the perovskite electrode material.

The results obtained for the specific capacitance of SrFeO_3_, SrCoO_3_ and SrFe_0.5_Co_0.5_O_3_ from CV and GCD curves show that in low scanning rates and high current densities, the specific capacitances of SrFeO_3_ are higher than those of other compounds, which could indicate the existence of the proper oxygen vacancy in the structure of this perovskite as well as the proper stability of these active points in the high current density. The partial substitution made has a negative effect on the specific capacitance of SrFeO_3_. This means that the oxygen vacancy concentration in SrFeO_3_ decreased after the partial substitution of Co at B site of SrFeO_3_.

Table 3 shows the specific capacitance obtained for different compounds that have been studied as electrode material for supercapacitor. As can be seen, perovskite compounds have high specific capacitance. The main advantage of these compounds is its proper stability during redox cycles, which causes a better preservation of the specific capacitance of the perovskite supercapacitor. The compounds synthesized in this study, which have been studied as two electrodes, in other words in the symmetric cell of the supercapacitor, have specific capacitance suitable for the supercapacitor. Nevertheless, it is still possible to increase their specific capacitance with a substitution and finding the right percentage combination of A and B site elements.

## 4. Conclusion

In this paper, three electrode materials including SrCoO_3_, SrFeO_3_ and SrCo_0.5_Fe_0.5_O_3_, were prepared to be used as supercapacitor electrode candidates; and the effect of partial substitution on electrochemical properties was investigated. The specific capacitance obtained for the SrCoO_3_, SrFeO_3_ and SrCo_0.5_Fe_0.5_O_3_ samples at the scan rate of 10 mV s^−1^ was 68.639, 101.687, and 60.912 F g^−1^, respectively, also similar to these results with a slight difference in the high current density. It was observed that accordingly SrFeO_3_ showed higher specific capacitance at low scan rates and higher current densities than other samples. Therefore, the partial substitution of Co at the B site of SrFeO_3_ in low scanning rates and high current densities has a negative effect on the specific capacitance and reduces it. This means that partial substitution reduces oxygen vacancy concentration as a charge carrier in the SrFeO_3_ structure. These observations were in argument with the results of EIS, where the SrFeO_3_ had the highest R_ct_ indicating slower charge transfer inside the electrode material, which decreases the contribution of faradaic currents in overall current density. The CV plats also confirmed this observation with a more rectangular shape of CVs for SrFeO_3_.

## Figures and Tables

**Table 1:** The specific capacitances of SrCoO_3_, SrFeO_3_ and SrCo_0.5_Fe_0.5_O_3_ at different scan rate.

Scan rate (mV/s)	Specific capacitance (F g^−1^)		
	SrCoO_3_	SrFeO_3_	SrCo_0.5_Fe_0.5_O_3_
10	68.639	101.687	60.912
20	26.157	51.667	43.449
50	7.646	26.108	28.99

**Table 2:** The specific capacitances of SrCoO_3_, SrFeO_3_ and SrCo_0.5_Fe_0.5_O_3_ at different current densities.

Current density (A g^−1^)	Specific capacitance (F g^−1^)		
	SrCoO_3_	SrFeO_3_	SrCo_0.5_Fe_0.5_O_3_
2	258.88	155.66	219.00
3	172.33	110.44	139.83
4	97.11	114.44	57.77

**Table 3:** The comparison different electrode materials used in supercapacitor.

Electrode materials	Specific capacitance	Electrolyte	Ref.
CNTs/MnO_2_	1229 F g^−1^ at 1 A g^−1^	1 mol L^−1^ Na_2_SO_4_	[44]
ZnO/MnO_x_	556 F g^−1^ at 1 A g^−1^	1 mol L^−1^ Na_2_SO_4_	[45]
CoO/NiO-Cu@CuO	2035 mF cm^−2^ at 2 mA cm^−2^	Solid-state PVA/KOH hydrogel	[46]
CNT-Fe_3_O_4_	373 F g^−1^ at 10 mV s^−1^	1 mol L^−1^ Na_2_SO_4_	[47]
LaCrO_3_	1268 F g^−1^ at 2 A g^−1^	Neutral LiCl aqueous	[48]
SrCo_0.9_Nb_0.1_O_3−δ_	773.6 F g^−1^ at 0.5 A g^−1^	Aqueous KOH	[37]
SrCoO_3_	258.88 F g^−1^ at 2 A g^−1^	1 mol L^−1^ KOH	This work
SrFe_0.5_Co_0.5_O_3_	219 F g^−1^ at 2 A g^−1^	1 mol L^−1^ KOH	This work
SrFeO_3_	114.44 F g^−1^ at 4 A g^−1^	1 mol L^−1^ KOH	This work
